# Are Mixed-Halide Ruddlesden–Popper Perovskites Really Mixed?

**DOI:** 10.1021/acsenergylett.2c01967

**Published:** 2022-10-31

**Authors:** Stefano Toso, Irina Gushchina, Allen G. Oliver, Liberato Manna, Masaru Kuno

**Affiliations:** † Department of Chemistry and Biochemistry, University of Notre Dame, Notre Dame, Indiana46556, United States; ‡ International Doctoral Program in Science, Università Cattolica del Sacro Cuore, 25121Brescia, Italy; § Department of Nanochemistry, Istituto Italiano di Tecnologia, Via Morego 30, 16163Genova, Italy; ∥ Department of Physics, University of Notre Dame, Notre Dame, Indiana46556, United States

## Abstract

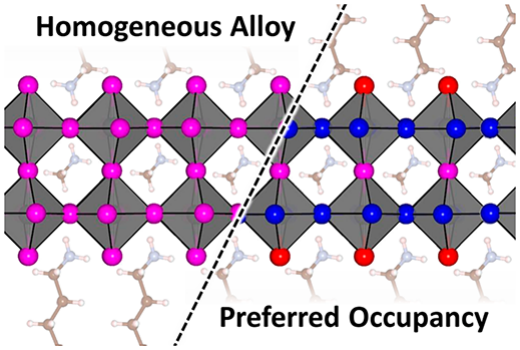

Mixing bromine and iodine within lead halide perovskites is a common strategy to tune their optical properties. This comes at the cost of instability, as illumination induces halide segregation and degrades device performances. Hence, understanding the behavior of mixed-halide perovskites is crucial for applications. In 3D perovskites such as MAPb(Br_*x*_I_1–*x*_)_3_ (MA = methylammonium), all of the halide crystallographic sites are similar, and the consensus is that bromine and iodine are homogeneously distributed prior to illumination. By analogy, it is often assumed that Ruddlesden–Popper layered perovskites such as (BA)_2_MAPb_2_(Br_*x*_I_1–*x*_)_7_ (BA = butylammonium) behave alike. However, these materials possess a much wider variety of halide sites featuring diverse coordination environments, which might be preferentially occupied by either bromine or iodine. This leaves an open question: are mixed-halide Ruddlesden–Popper perovskites really mixed? By combining powder and single-crystal diffraction experiments, we demonstrate that this is not the case: bromine and iodine in RP perovskites preferentially occupy different sites, regardless of the crystallization speed.

Ruddlesden–Popper (RP) lead halide perovskites are a class of two-dimensional (2D) semiconductors that have recently gained relevance as promising candidates for optoelectronic and photovoltaic applications.^1−6^ Described by the formula L_2_A_*n*–1_Pb_*n*_X_3*n*+1_,^7^ they consist of *n* two-dimensional layers of corner-sharing [PbX_6_]^4–^ octahedra (X = Cl, Br, I) held together by isotropic cations (A = Cs^+^, methylammonium (MA), formamidinium (FA), ...). Individual layers, in turn, are separated by long-chain ammonium cations such as butylammonium (L = BA) or phenylethylammonium (L = PEA).^3^

A demand for band gap tuning, electronic structure engineering, and integration into three-dimensional (3D) perovskite solar cells is now extending interest to mixed bromide–iodide RP perovskites, L_2_A_*n*–1_Pb_*n*_(Br_*x*_I_1–*x*_)_3*n*+1_, where *x* indicates the bromine fraction. Recent reports confirm that mixing halides in RP perovskites offers additional control over their optical properties^8^ and enables the use of a wider variety of L cations in comparison to single-halide RP perovskites.^9^ Other studies have demonstrated the creation of vertical and horizontal heterojunctions, obtained by stacking premade RP sheets^10^ or by exploiting halide-diffusion reactions.^11^

Despite progress in the area, applications of 2D mixed-halide perovskites are limited by photoinduced anion segregation. Recent investigations into their photostability highlight a tendency of halide anions to migrate within crystals under illumination,^12−14^ similar to what is observed in 3D mixed-halide APb(Br_*x*_I_1–*x*_)_3_ perovskites.^12,13^ In this regard, photoinduced anion segregation appears to be an intrinsic instability of lead halide perovskites as a whole.

As early studies shed light on the behavior of mixed-halide RP perovskites under external stimuli, questions have arisen about their properties when at rest. Indeed, while the structures of 3D mixed-halide perovskites are well described as halide alloys,^15,16^ there are reasons to believe that the situation is more complex in 2D mixed-halide RP perovskites. Compared to 3D perovskites, RP structures offer a greater diversity of halide crystallographic sites. Of these, some sites are embedded deep within inorganic layers (central sites, Ct), while others protrude directly into the organic cation layers (apical sites, Ap) or alternatively create extended horizontal networks that form the inorganic layers (equatorial sites, Eq).

This diversity of coordination environments may therefore promote the occupation of certain sites by different anions, by virtue of differences in ionic radii or interaction affinities with cations. Recently reported theoretical predictions bring arguments to support such preferential positioning of halides in (PEA)_2_Pb(Br_*x*_I_1–*x*_)_4_.^8^ These results are corroborated by single-crystal X-ray diffraction studies on (*ter*-BA)_2_Pb(Br_0.5_I_0.5_)_4_.^9^ The same effect has also been reported for other layered metal halides such as (MA)_2_Cu(Cl_*x*_Br_1–*x*_)_4_.^17−19^ This poses a question: are lead-based mixed-halide RP perovskites really mixed alloys?

To answer this question, we investigate the structure of bilayer (BA)_2_MAPb_2_(Br_*x*_I_1–*x*_)_7_ RP perovskites. We have chosen bilayers (*n* = 2) because they offer a wider variety of halide crystallographic sites compared to *n* = 1 monolayers, where sites embedded deep in the inorganic layers (Ct) are missing due to insufficient thickness. Samples have been synthesized by adapting reported methods,^7,10^ with details being provided in the Supporting Information. Briefly, for each sample a stock solution is prepared by dissolving methylammonium, butylammonium, and lead iodides and bromides in a hot mixture of concentrated HI, HBr, and H_3_PO_2_. Precursor ratios, detailed in Table S1, determine the resulting sample stoichiometry. Once solubilized, solutions are cooled to 35–40 °C, causing the precipitation of RP perovskite powders that are used for powder X-ray diffraction (PXRD) analyses. Alternatively, RP crystals are grown on substrates by drop-casting the warm solution. This initiates nucleation and growth, which is then halted by drying substrates with a paper tissue. The procedure yields platelet-shaped crystals with lateral sizes of ∼10–100 μm (Figure 1a), which are used for compositional analysis and optical microscopy. Their habit is remindful of the RP structure,^7^ having wide and flat [010] facets, laterally terminated by perpendicular [101] and [101̅] facets.

**Figure 1:**
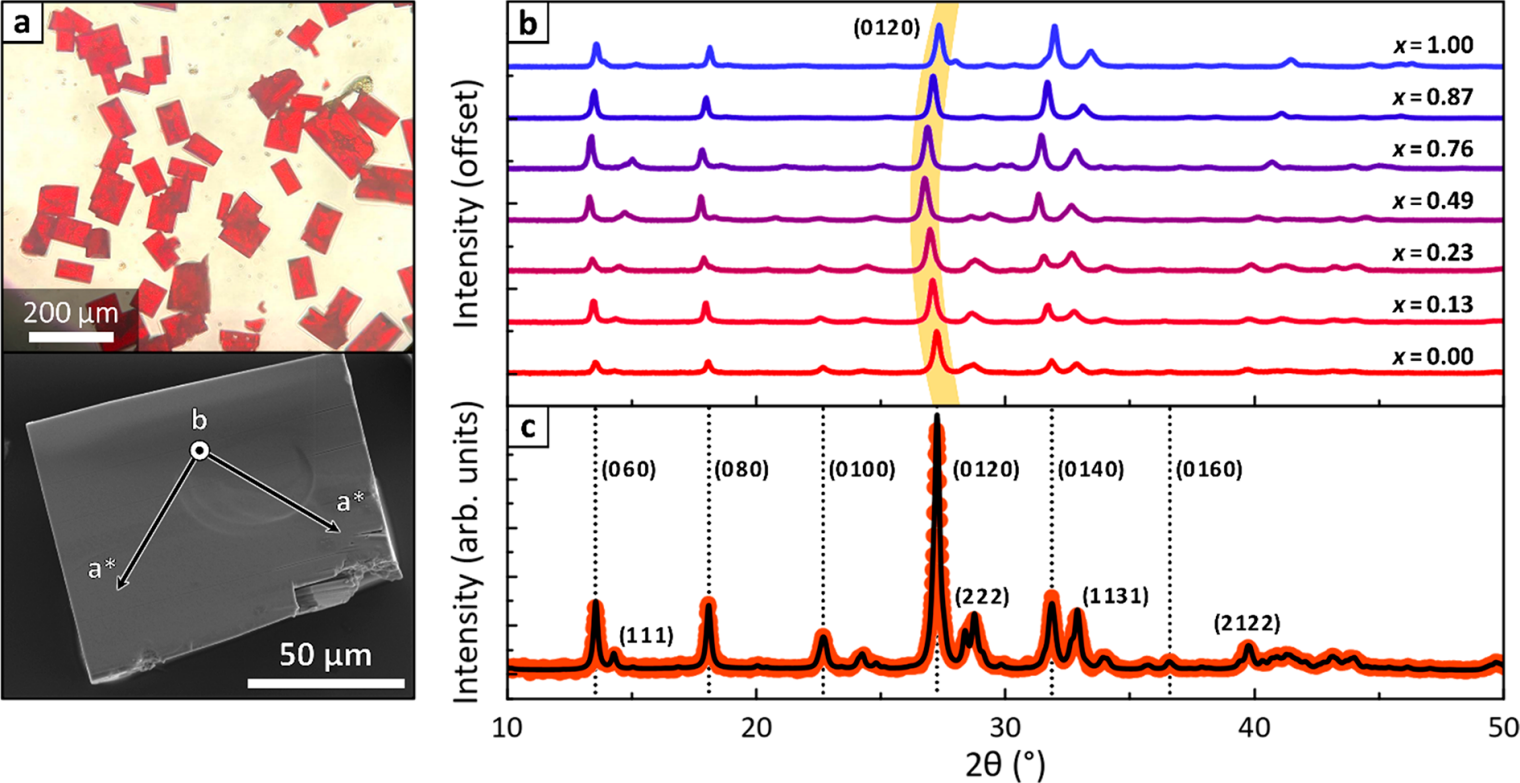
PXRD characterization of (BA)_2_MAPb_2_(Br_*x*_I_1–*x*_)_7_. (a) (top) Optical microscopy image of (BA)_2_MAPb_2_(Br_*x*_I_1–*x*_)_7_ crystals. (bottom) SEM image of a representative crystal, with overlaid lattice vectors. (b) PXRD patterns of (BA)_2_MAPb_2_(Br_*x*_I_1–*x*_)_7_ samples. Highlighted are the (0120) peaks, whose shift toward lower angles highlights the anomalous unit cell expansion along the *b* cell axis for mixed-halide compositions. (c) Le Bail profile fit of the (BA)_2_MAPb_2_I_7_ PXRD pattern. Vertical dotted gray lines indicate the family of (02*k*0) peaks typical of RP perovskites. Some (*hkh*) peaks, which ensure a reliable determination of the a* parameter, have also been indexed.

Sample compositions have been verified using scanning electron microscopy–energy dispersive X-ray spectroscopy (SEM-EDXS). All mixed-halide specimens were found to be significantly richer in iodine than expected from their stock solution feed ratios. This indicates a tendency of RP structures to incorporate iodine over bromine. A calibration curve, relating measured experimental compositions to those of starting precursor solutions, was therefore constructed (see Figure S1 in the Supporting Information). In what follows, sample compositions are labeled using *x*_tot_ = Br/[Br + I], where Br and I are the measured halide atomic fractions: *x*_tot_ = 0 stands for pure-iodide samples, while *x*_tot_ = 1 stands for pure-bromide samples.

All samples were characterized via PXRD (Figure 1b). Their diffraction patterns feature a series of intense and periodic (02*k*0) peaks, typical of RP perovskites (see also Figure S2 in the Supporting Information).^6,7^ A Le Bail profile fit was performed on all patterns (Figure 1c shows it for *x* = 0; other fits are available in Figure S3 in the Supporting Information) to trace how unit cell parameters change with halide composition. Because RP perovskites are pseudotetragonal materials, most reflections that differentiate *a* and *c* (i.e., *h* ≠ *l*) are weak and overlap strongly. This makes their individual measurements unreliable. In contrast, a pseudotetragonal a* parameter that represents an average of *a* and *c* is easily determined, based on strong and well-resolved (*hkh*) reflections.

As expected, a* decreases linearly from a* = 8.92 Å for *x* = 0 to a* = 8.33 Å for *x* = 1 (Figure 2a). The *b* parameter, however, does not change linearly with halide composition (Figure 2b). Rather, it first rises and then falls, reaching a maximum at *x*_tot_ = 0.5 (*b* = 39.9 Å). This behavior is foreshadowed in Figure 1b by the marked shift of (02*k*0) peaks toward lower angles. Together, a* and *b* result in a monotonic decrease of the unit cell volume (*V*_cell_ = a*^2^*b*) as *x*_tot_ increases from 0 to 1 (Figure 2c). Only a mild positive inflection is seen due to the nonlinear behavior of *b*.

**Figure 2:**
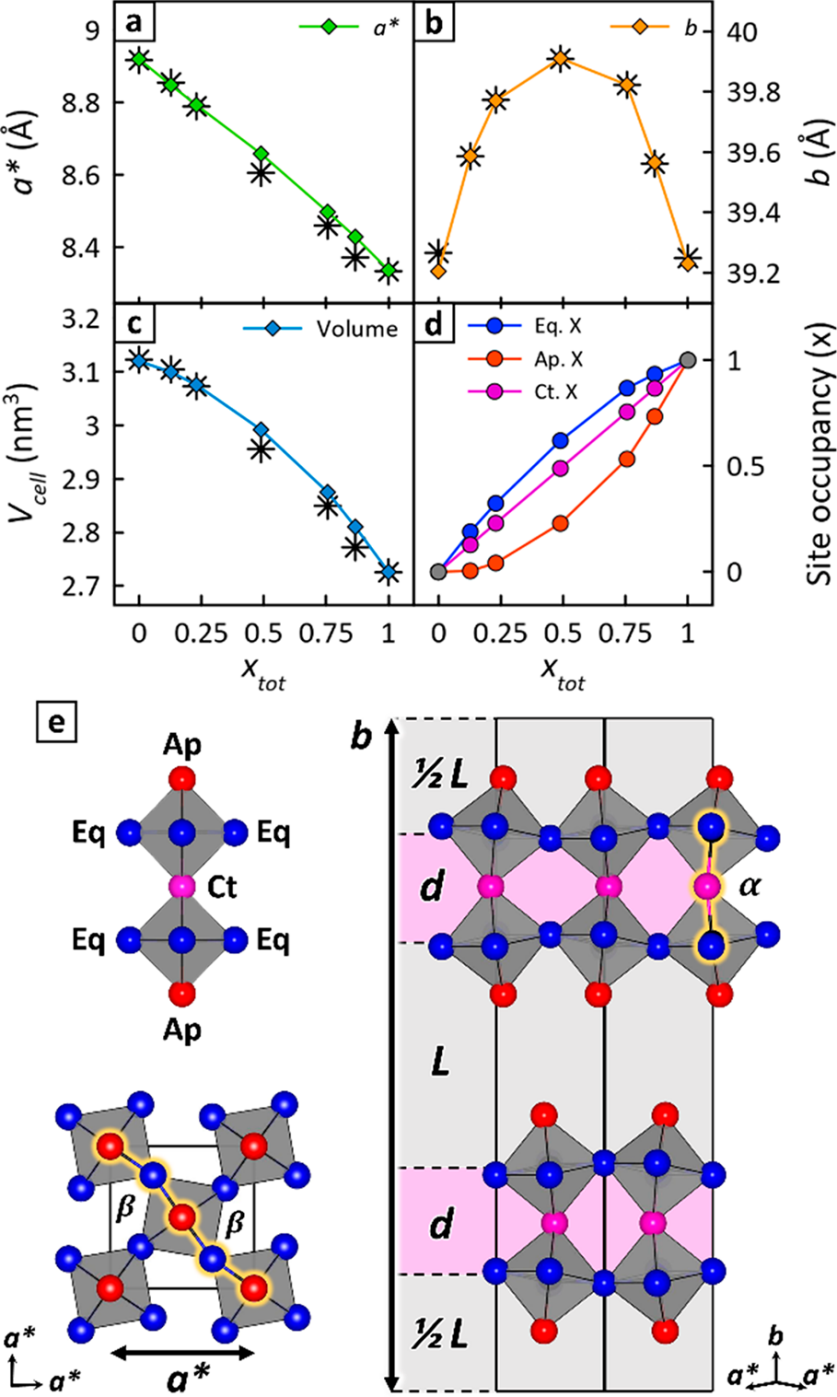
Impact of halide distribution on (BA)_2_MAPb_2_X_7_ unit cell parameters. (a–c) Experimental RP unit cell parameters (black asterisks) and model predictions (colored lines). (d) Halide distribution in apical (Ap), equatorial (Eq), and central (Ct) sites, corresponding to parameters predicted in (a)–(c). (e) Visual representation of the model, showing how a* and *b* are functions of bond lengths and tilt angles, and of the organic cation spacing *L*. The inorganic layer thickness, *d,* is defined in eq 2. In (d) and (e), red, blue, and purple identify Ap, Eq, and Ct sites, respectively.

Such an anomalous dependence of *b* on the halide composition suggests that lead-based mixed-halide RP perovskites *do not* behave as ideal alloys when it comes to placing I^–^ and Br^–^ in the structure. To rationalize this behavior, we therefore construct a geometric model (Figure 2) that relates RP unit cell parameters to the distribution of halide anions in different crystallographic sites (Ap, Ct, and Eq). The model is semiquantitative and rationalizes the observed PXRD structural trends summarized in Figure 2a–c.

In the model, unit cell parameters a* and *b* are functions of [Pb–X] bond lengths, of horizontal and vertical octahedra tilt angles (α and β), and of the organic cation layers thickness *L* (eqs 1 and 2). All parameters in eqs 1 and 2 depend on the halide occupation of Ap, Eq, and Ct sites, which are related to each other and to experimentally established sample halide compositions, *x*_tot_, via the material stoichiometry (eq 3).
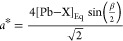
1
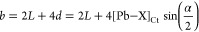
2
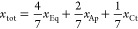
3

Lead–halide bond lengths ([Pb–X]) are modeled as linear combinations of limiting [Pb–Br] and [Pb–I] bond distances, as measured by single-crystal X-ray diffraction (SCXRD) on (BA)_2_MAPb_2_Br_7_ and (BA)_2_MAPb_2_I_7_, weighted by the fractional occupation of respective halide sites (Table S2). In eqs 1 and 2, the subscripts Eq and Ct identify the site involved in each [Pb–X] bond and the bond itself. Similarly, α and β are estimated from limiting values determined by SCXRD (Table S3). We additionally note that octahedral tilting modes adopted by pure-bromide and pure-iodide structures are incompatible.^7,20^ We therefore assume that a transition structure at *x*_tot_ = 0.5 does not experience octahedral tilt in any direction. This assumption is later proven correct. Finally, *L* is estimated by assuming that the volume occupied by butylammonium chains remains constant in all structures (see the Supporting Information and Table S4 for further discussion). A detailed description of the model and explanations of how [Pb–X], α, β, and *L* depend on the halide composition of Ap, Eq, and Ct sites can be found in Figure S4 in the Supporting Information.

The only free variable in the model is *x*_Eq_. Indeed, given the similarity to bulk halide sites in 3D perovskites, Ct sites are assumed to be occupied by I^–^ and Br^–^ with no preference (i.e., *x*_Ct_ ≈ *x*_tot_). Consequently, *x*_Ap_ is a function of *x*_Eq_ (*x*_Ap_ = 3*x*_tot_ – 2*x*_Eq_, from eq 3). With this, PXRD-extracted *b* values (Figure 2b) are fit using the model by varying *x*_Eq_. The fit simultaneously yields model-predicted a* and *V*_cell_ values that can be further compared with experiments (Figure 2a,c) to assess the model’s overall performance.

Solid colored lines in Figure 2 reveal that the model captures the linear trend of a* in Figure 2a, the peaked behavior of *b* in Figure 2b, and the mild positive inflection of *V*_cell_ in Figure 2c as *x*_tot_ increases from 0 to 1. These results correspond to a marked excess of iodine in unit cell apical positions and bromine in equatorial positions. The trend is observed over the entire (BA)_2_MAPb_2_(Br_*x*_I_1–*x*_)_7_ composition range (Figure 2d). As a point of reference, for *x*_tot_ = 0.5 the halide distribution predicted by the model is Ap = 23% Br, Eq = 62% Br, and Ct = 50% Br.

Overall, these results demonstrate that the anisotropic unit cell expansion observed by PXRD arises from a preferential positioning of halide anions. Such an expansion is rationalized as the combination of two effects. First, the prevalence of larger I^–^ ions in apical positions results in an elongation of [Pb–X] bonds parallel to the unit cell *b* axis. Second, the prevalence of smaller Br^–^ ions in equatorial positions shrinks the lattice on the *a–c* plane. This laterally compresses the BA cations and forces them to expand along the *b* axis. What results is an increase of the interlayer distance, *L*. This conclusion is corroborated by SCXRD-solved structures of (BA)_2_MAPb_2_Br_7_ and (BA)_2_MAPb_2_I_7_, where *L* = 13.49 Å in the former and *L* = 13.15 Å in the latter. As both effects are solely dependent on [Pb–-X] bond distances, the behavior is expected to be general across RP structures made using different organic cations.

Furthermore, the anisotropic expansion along *b*, measured as a bowing in the position of PXRD (02*k*0) reflections, appears to be a reliable approach for detecting the preferential positioning of halide anions in mixed-halide RP structures. Indeed, further simulations (Figure S5) indicate that the *b* parameter could grow as much as +4.3% if halides displayed full preferentiality for apical and equatorial sites and would instead decrease by −1.3% if halides were randomly alloyed (with the reference value being the average *b* parameter for pure-halide structures, 39.26 Å).

To support and validate our model, single-crystal specimens of (BA)_2_MAPb_2_I_7_, (BA)_2_MAPb_2_Br_7_, and mixed-halide *x*_tot_ = 0.5 (BA)_2_MAPb_2_(Br_0.5_I_0.5_)_7_ were grown by slowly cooling precursor solutions and were subsequently analyzed by SCXRD. Data have been collected at room temperature to ensure that structure parameters (bond lengths and angles, unit cell parameters) remained consistent with those measured in PXRD experiments. This also prevented any thermal stress from altering the distribution of halide anions in structures. A detailed description of the SCXRD analysis can be found in the Supporting Information.

SCXRD results on pure-bromide and pure-iodide structures corroborate earlier literature reports.^7,20^ They confirm that (BA)_2_MAPb_2_Br_7_ and (BA)_2_MAPb_2_I_7_ crystallize in the *Ccc*2 and *Ccm*2_1_ space groups, respectively (Figure 3a). (BA)_2_MAPb_2_Br_7_ adopts an octahedral tilting mode, denoted (00Φ_*z*_) in an Aleksandrov notation (Figure 3b, top),^21^ with pairs of octahedra rotated in antiphase around the *b* axis by ∼10°. (BA)_2_MAPb_2_I_7_ adopts a (Φ_1_Φ_1_0) tilting mode (Figure 3b, bottom), with the two combined tilts resulting in an antiphase rotation of octahedra around the *c* axis (8.6°). In contrast, the *x* = 0.5 (BA)_2_MAPb_2_(Br_0.5_I_0.5_)_7_ specimen crystallizes in the higher symmetry space group *Fmmm* (Figure 3a, middle). Consistent with assumptions in our geometric model, the transition between these two incompatible tilting modes suppresses any tilt (Figure 3b, middle), a condition denoted (000). This conclusively demonstrates that a coexistence of I^–^ and Br^–^ in 2D RP structures can cause major modifications to their crystal symmetry.

**Figure 3:**
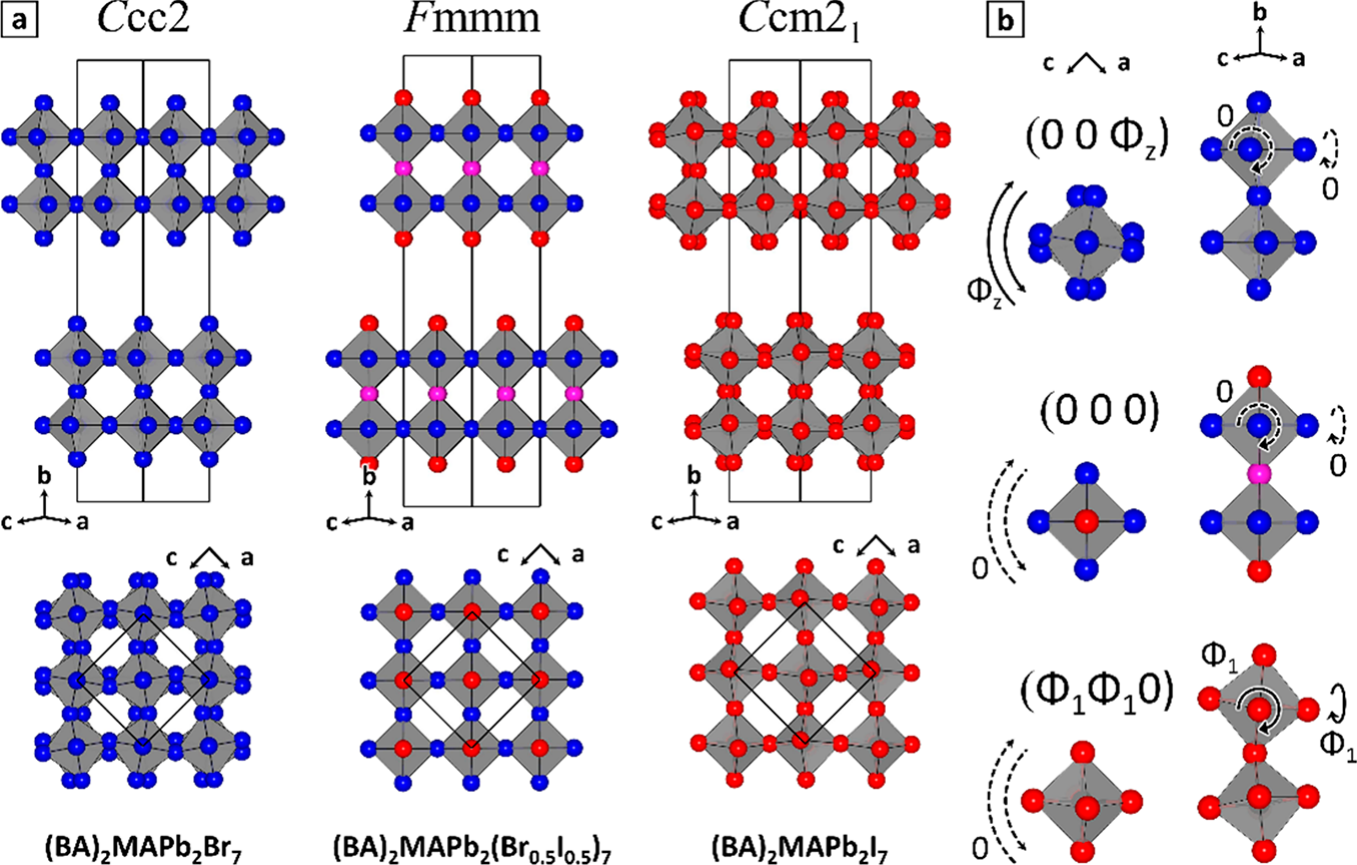
Structures of pure- and mixed-halide (BA)_2_MAPb_2_X_7_ samples. (a) Crystal structures of (BA)_2_MAPb_2_Br_7_ (left), (BA)_2_MAPb_2_(Br_0.5_I_0.5_)_7_ (middle), and (BA)_2_MAPb_2_I_7_ (right) as solved by SCXRD. (top) (101) projection. (bottom) (010) projection. Halide site color code: blue, Br (exclusive or prevalent); red, I (exclusive or prevalent), purple, comparable Br and I fractions. Organic cations have been omitted for clarity. (b) Representation of octahedral tilting modes in structures, as described by an Aleksandrov notation.^21^

Crucially, a bond length analysis of the *x*_tot_ = 0.5 (BA)_2_MAPb_2_(Br_0.5_I_0.5_)_7_ sample indicates that Ap positions are mostly occupied by iodine (Ap = 29% Br, 71% I). Equatorial positions are instead richer in bromine (Eq = 74% Br, 26% I), while Ct positions are occupied more homogeneously (Ct = 42% Br, 58% I), corresponding to a crystal composition of *x*_tot_ = 0.56 (Table S5). Those results are very close to the halide distribution predicted by the geometric model for *x* = 0.5. A complementary analysis of halide site occupation via electron densities confirms the same trend and suggests a more marked preference of apical and central sites for iodine (Ap, 7% Br, 93% I; Ct, 37% Br, 63% I; Eq, 64% Br, 36% I). The extracted crystal composition is *x*_tot_ = 0.44, again compatible within error with the target *x*_tot_ = 0.5 composition (Table S6).

Overall, both PXRD and SCXRD data point to a preferential positioning of I^–^ and Br^–^ anions in lead-based mixed-halide RP structures. Discrepancies in determined occupancies likely stem from a combination of model assumptions (e.g., *x*_Ct_ ≈ *x*_tot_), approach biases (bond lengths vs electron density), and crystallization speeds. The last point is especially relevant, given that sample preparation methods might influence the crystallization dynamics and therefore the distribution of anions within mixed-halide RP structures. However, both PXRD and SCXRD showed clear evidence of preferential halide positioning and crystallization occurred over minutes and hours, respectively. Therefore, the question arises whether *faster* crystallization conditions might favor a more homogeneous halide distribution. To address this question, we have collected XRD patterns of *n* = 2 (BA)_2_MAPb_2_(Br_*x*_I_1–*x*_)_7_ and *n* = 1 (BA)_2_Pb_2_(Br_*x*_I_1–*x*_)_4_ RP perovskite thin films prepared by spin coating (Figures S6 and S7). The latter *n* = 1 specimens are of particular interest, given extensive prior investigations of these materials.^8,9,12,13^ Details of the thin-film spin-coating synthesis have been provided in the Supporting Information.

For both *n* = 1 and *n* = 2 thin films, Figure 4 shows clear nonlinear *b* dependences with halide composition. This confirms that halide sites in mixed-halide RP perovskites are preferentially occupied even under the fast crystallization conditions often adopted for fabricating samples and devices.^1−3,9,12−14,22^ Notably, both *n* = 1 and *n* = 2 samples demonstrate comparable maximum expansions along *b* (*n* = 1, +1.58%; *n* = 2, +1.73%).

**Figure 4:**
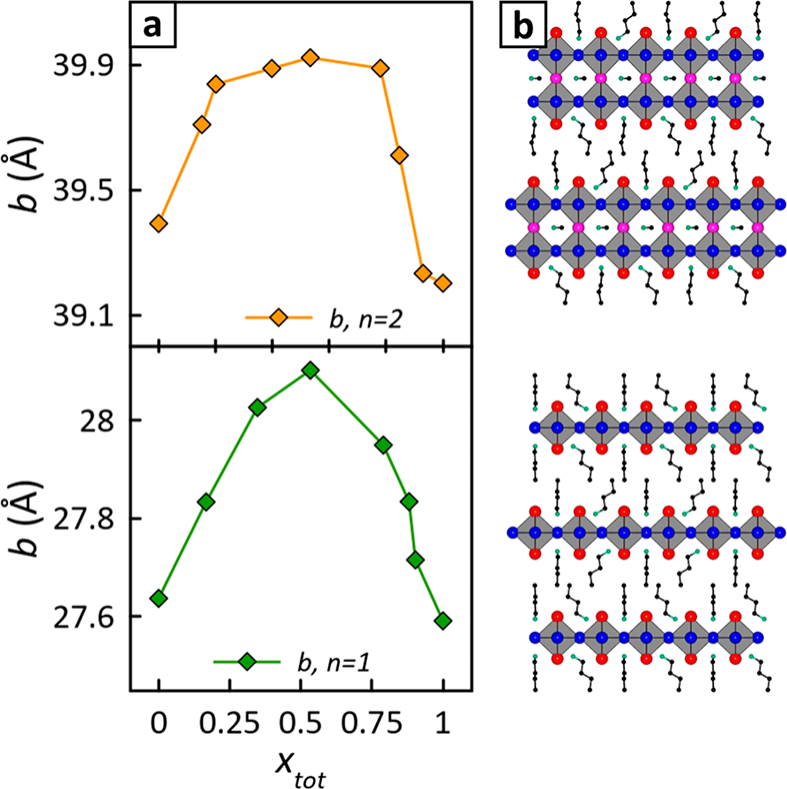
Cell parameter *b* in thin films. (a) Experimental *b* parameter measured on *n* = 2 (top) and *n* = 1 (bottom) thin films prepared by spin coating. (b) Structure schemes of *n* = 2 (BA)_2_MAPb_2_X_7_ (top) and *n* = 1 (BA)_2_PbX_4_ (bottom) RP perovskites.

To conclude, the common assumption that anions in mixed-halide perovskites are homogeneously distributed at the unit cell level, which holds for 3D perovskites because their halide sites share comparable chemical environments,^15,16,23−25^ does not apply to RP layered perovskites. In RP perovskites, instead, I^–^ anions preferentially occupy apical sites that are closest to long-chain organic cations, while Br^–^ anions prefer to reside in equatorial sites, surrounded by lead cations. This preferential positioning occurs regardless of the crystallization speed and is likely due to the different ionic radii of iodine and bromine. Our conclusions are supported by recent reports on lead-based mixed-halide RP perovskites^9,17,26^ and appear to be part of a broader trend that extends beyond the domain of lead-based materials, wherein structural anisotropy emerges to inhibit halide alloying.^17−19,27,28^

Although the effect of such an inhomogeneous halide distibution on the optoelectronic properties of mixed-halide RP perovskites has yet to be fully investigated, there are reasons to believe that it might not be negligible. Indeed, we demonstrated that mixed-halide RP samples can adopt altered symmetry and octahedral tilting modes compared to pure-halide RP specimens. This might affect the orbital overlap and thus the electronic structure of these materials. Moreover, being aware of preferential anion occupation in mixed-halide RP structures might assist in better understanding the recent observations of anion photosegregation in these materials.

## Supporting Information Available

(PDF) C(CIF) The Supporting Information is available free of charge at https://pubs.acs.org/doi/10.1021/acsenergylett.2c01967.

Synthesis and characterization methods, oriented- and powder-XRD patterns of (BA)_2_MAPb_2_(Br_*x*_I_1–*x*_)_7_ polycrystalline samples and Le Bail fits of the latter, outline of the RP structure geometric model, discussion of SCXRD analyses, additional characterization of spin-coated thin films (PDF)
Crystallographic information files of the (BA)_2_MAPb_2_Br_7_, (BA)_2_MAPb_2_I_7_, and the (BA)_2_MAPb_2_(Br_0.5_I_0.5_)_7_ structures solved by SCXRD (ZIP)
