# Supplementary material for: Are Mixed-Halide Ruddlesden–Popper Perovskites Really Mixed?
Source: ACS Energy Lett. 2022 Oct 31;7(12):4242–7. doi: 10.1021/acsenergylett.2c01967 (PMC9748757; doi:10.1021/acsenergylett.2c01967)
Supplement: Supplementary file 1 — nz2c01967_si_001.pdf [file nz2c01967_si_001.pdf]

Supporting Information for

Are Mixed-Halide Ruddlesden-Popper Perovskites Really Mixed?

Stefano Toso[1,2,3,], Irina Gushchina[1], Allen G. Oliver[1], Liberato Manna[3], Masaru Kuno[1,4]*

1) Department of Chemistry and Biochemistry, University of Notre Dame, Notre Dame, IN 46556, USA
2) International Doctoral Program in Science, Università Cattolica del Sacro Cuore, 25121 Brescia, Italy
3) Department of Nanochemistry, Istituto Italiano di Tecnologia, Via Morego 30, 16163 Genova, Italy
4) Department of Physics, University of Notre Dame, Notre Dame, IN 46556, USA

E-mail: stefano.toso@iit.it

Methods

Chemicals

Lead bromide ($PbBr_2$, >98% trace metal basis), lead iodide (PbI_2, 99.999% trace metal basis), methylammonium bromide [CH_6NBr (MABr), 98%], methylammonium iodide [CH_6NI (MAI), 98%], butylammonium bromide [$C_4H_{12}NBr$ (BABr), 98%], butylammonium iodide [$C_4H_{12}NI$ (BAI), 98%], hydrobromic acid (HBr, 48% aq.), hydroiodic acid (HI, 57% aq.), and hypophosphorous acid (H_3PO_2, 99% aq.) were purchased from Sigma-Aldrich. All chemicals were used without further purification.

Synthesis of $(BA)_2MAPb_2X_7$ samples

In brief, MABr, MAI, BABr, BAI, $PbBr_2$ and PbI_2 were weighed inside 8 mL glass vials (MA = methylammonium, BA = butylammonium). To this, known amounts of concentrated aqueous solutions of HBr, HI and H_3PO_2 were added. **Table S1** provides specific amounts of each reagent used.

Table S1. Stock solution compositions for $(BA)_2MAPb_2(Br_xI_{1-x})_7$ samples.

Feed Ratio (x_{feed})	Final Composition (x_{tot})	BABr (g)	BAI (g)	MABr (g)	MAI (g)	$PbBr_2$ (g)	PbI_2 (g)	HBr (mL)	HI (mL)	H_3PO_2 (mL)
**0**	**0**	---	0.173	---	0.099	---	0.544	---	1.818	0.182
**0.2**	**0.034**	0.026	0.138	0.014	0.079	0.087	0.435	0.321	1.497	0.182
**0.4**	**0.046**	0.053	0.104	0.028	0.059	0.173	0.326	0.661	1.157	0.182
**0.5**	**0.079**	0.066	0.086	0.035	0.049	0.217	0.272	0.839	0.979	0.182
**0.6**	**0.128**	0.079	0.069	0.042	0.039	0.260	0.218	1.023	0.795	0.182
**0.7**	**0.230**	0.093	0.052	0.049	0.030	0.303	0.163	1.212	0.606	0.182
**0.8**	**0.489**	0.106	0.035	0.056	0.020	0.346	0.109	1.408	0.410	0.182
**0.90**	**0.756**	0.119	0.017	0.062	0.010	0.390	0.054	1.610	0.209	0.182
**0.95**	**0.867**	0.126	0.009	0.066	0.005	0.411	0.027	1.713	0.105	0.182
**1.0**	**1**	0.132	---	0.069	---	0.433	---	1.818	---	0.182

Powders: Vials were sealed and heated to 130 °C on a hotplate until their contents became limpid light-yellow solutions. Gentle shaking was used to speed up this process. Once solutions turned clear, vials were transferred to a thermostatic water bath, maintained at temperatures between 35-40 °C. Crystalline RP precipitates were obtained within a few minutes of immersion. Gentle shaking was used to speed up this process.

Crystals on substrates: To synthesize RP crystals on substrates, 50 µL of warm (mother liquor) solutions were drop cast onto flat glass or silicon substrates. Crystal nucleation was triggered on cooling and was observed through the formation of yellow-to-orange colored films. Crystal growth was halted by gently pressing paper tissues onto substrates to wick up any excess mother liquor.

PXRD specimens. To retrieve powders for PXRD analyses, vials were cooled to room temperature and were centrifuged to compact powders onto their bottoms (2500 rpm, 5 minutes). Any supernatant was removed using a pipette. Wet solids were then recovered with a spatula and deposited onto filter paper. Samples were dried through gentle pressing with additional filter paper. Recovered solids were then transferred to a mortar and were gently ground with a pestle to randomize the orientation of RP crystallites. Powders were finally transferred into a cylindric sample holder (∼6 mm diameter ×3 mm depth) and were gently pressed to obtain flat pellets for PXRD analysis.

SCXRD samples. Single crystal specimens were prepared in a manner similar to that of powder samples. The main difference was that samples were cooled from 130 °C to room temperature with a controlled temperature descent of -2 °C per hour, using a programmable oven. Additionally, 40 mL vials were used to ease the manipulation of resulting single crystals. Produced specimens contained compact masses of mm-sized crystals, that were highly defective. Consequently, for SCXRD analyses thin shards of approximately 95 mm in lateral size were selected, based on the quality of their polarized light extinction.

Spin-coated films. Pure iodide and bromide precursor solutions were prepared from pure iodide and bromide RP perovskite powder samples, obtained as described above for PXRD specimens. The composition of n = 2 stock solutions is indicated in **Table S1**, while that for n = 1 samples follows: $(BA)_2PbBr_4$ = 0.349 g $PbBr_2$ + 0.085 g BABr + 1.818 ml HBr + 0.182 ml H_3PO_2; $(BA)_2PbI_4$ = 0.438 g PbI_2 + 0.111 g BAI + 1.818 ml HI + 0.182 ml H_3PO_2. The as-prepared RP perovskite powders were weighed and dissolved in anhydrous dimethylformamide (DMF), using suitable volumes to produce equimolar solutions of n = 1 $(BA)_2PbBr_4$ and $(BA)_2PbI_4$ and n = 2 $(BA)_2MAPb_2Br_7$ and $(BA)_2MAPb_2I_7$. To obtain mixed-halide $(BA)_2PbX_4$ and $(BA)_2MAPb_2X_7$ thin film specimens, precursor solutions were pre-mixed in the desired stoichiometric ratio and coated onto plasma-treated glass coverslips.

Characterization

EDXS analysis. Compositional analysis was performed on a Thermo Prisma Environmental-SEM equipped with an EDXS probe. Crystalline samples were deposited onto silicon substrates and were measured without additional sputtering of conductive coatings. Compositional data were acquired in mapping mode on regions of interest, comparable in size with microcrystal (010) facets. Multiple microcrystals (N~5) were analyzed for each RP composition made. Average stoichiometries are provided in **Figure S1**. Spin coated film samples were analyzed in 3 different regions of the film to average the compositional analysis.

PXRD and Le Bail analysis. Powder XRD patterns were collected on a Bruker D8 Advance diffractometer, equipped with a Cu Kα source and a LynxEye pixel detector operating in a Bragg-Brentano geometry in reflection mode. PXRD patterns were analyzed by performing Le Bail fits using the program Profex.[1] Prior to analysis, manual background subtraction was performed to improve fit convergence. The Le Bail analysis does not assume any structural constraints on the intensities of diffraction peaks. Hence, it is ideal for refining unit cell parameters, but does not provide any structural information beyond this.

SCXRD analysis. SCXRD data were collected on a Bruker Apex II single-crystal diffractometer, equipped with a Mo-Kα source and Apex-II detector. Data were collected at room temperature using Apex-4 software. Subsequent data analysis and structure solution were performed using the software suite Olex2.[2] Details of the refinements are provided in the "*Single-crystal X-ray diffraction results*" section below.

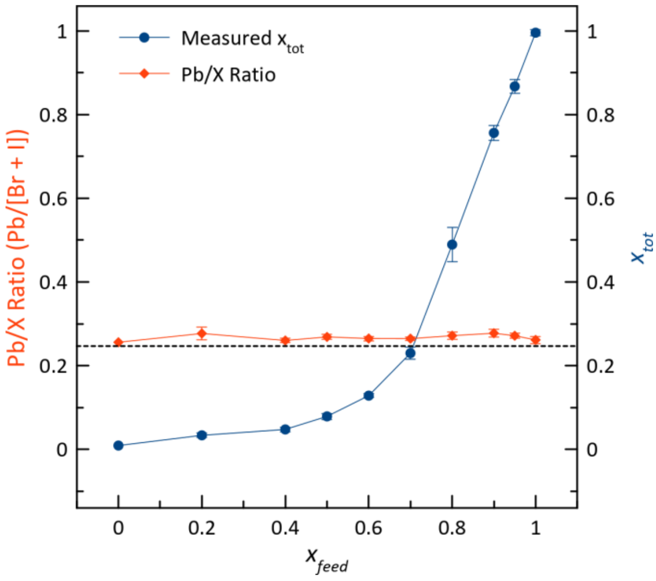

Figure S1. Composition calibration curve for $(BA)_2MAPb_2(Br_xI_{1-x})_7$ crystals. Blue trace: experimental halide composition plotted versus halide precursor feed ratio. Red trace: experimental Pb/X ratio as measured by SEM-EDXS. In both scales, x = Br/[Br+I] where Br and I are the atomic fractions of bromine and iodine introduced in precursor solutions (feed ratio) and measured experimentally by SEM-EDXS (measured). Solid blue and red lines are guides to the eye. The horizontal, dashed black line represents the ideal Pb/X value of 2/7 = 0.286, established by the stoichiometry of $(BA)_2MAPb_2X_7$. This line serves as a control to assess the reliability of SEM-EDXS analyses.

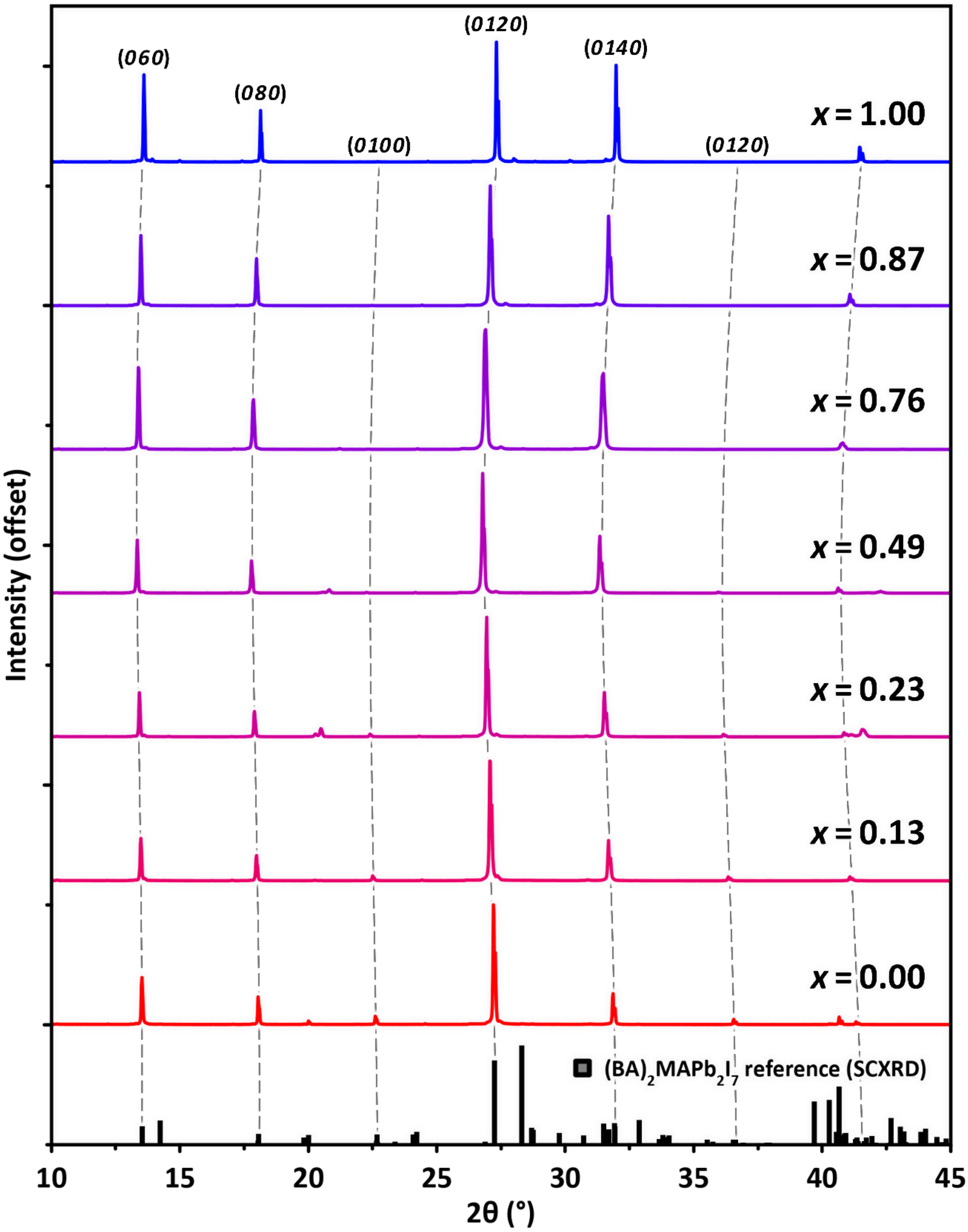

Figure S2. XRD patterns of $(BA)_2MAPb_2(Br_xI_{1-x})_7$ crystals oriented flat on substrates. Data were collected on samples prepared for SEM-EDXS analysis. All patterns show a series of intense and regularly spaced reflections typical of RP perovskite crystals laying with (*02k0*) planes parallel to the substrate. All peaks are captured by the same periodicity, indicating that only the n = 2 $(BA)_2MAPb_2(Br_xI_{1-x})_7$ phase is present, and no contaminant RP phases with n = 1 or n ≥ 3 were formed. The few, weak peaks not belonging to the periodic series come from misoriented crystals of $(BA)_2MAPb_2(Br_xI_{1-x})_7$ RP perovskites, as seen by comparison with the reference pattern (reference structure from this work).

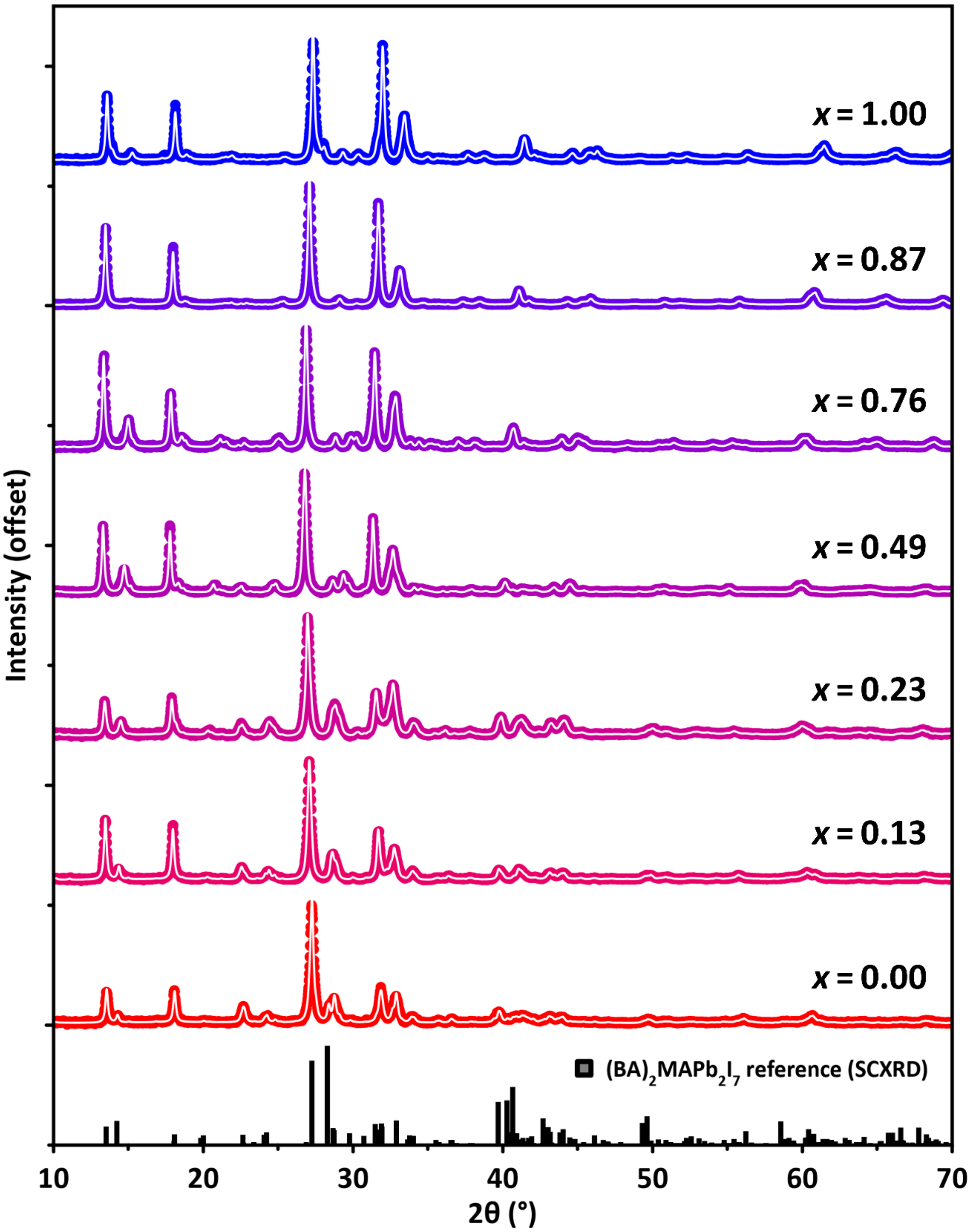

Figure S3. Le Bail fits of $(BA)_2MAPb_2(Br_xI_{1-x})_7$ PXRD patterns. Experimental PXRD patterns collected on $(BA)_2MAPb_2(Br_xI_{1-x})_7$ samples (colored markers) with Le Bail fit profiles superimposed (solid white lines). All peaks in the diffraction patterns could be indexed based on the expected $(BA)_2MAPb_2(Br_xI_{1-x})_7$ structures, indicating that all samples are phase-pure. The reference pattern is calculated based on the $(BA)_2MAPb_2I_7$ RP perovskite structure solved in this work by SCXRD.

RP structure geometric model

As discussed in the main text, RP unit cell parameters can be written as:

$$a^* = \frac{4[\mathrm{Pb{-}X}]_{\mathrm{Eq}} \sin\frac{\beta}{2}}{\sqrt{2}} \tag{S1}$$

$$b = 2L + 4[\mathrm{Pb} - \mathrm{X}]_{\mathrm{Ct}} \sin\frac{\alpha}{2} \tag{S2}$$

Where [Pb-X] is the Pb-X bond length, with subscripts indicating the relative halide site, *L* is the Pb-Pb vertical distance between neighboring $[PbX_6]^{4-}$ octahedra layers, α is the Pb-X-Pb bond angle between vertically stacked $[PbX_6]^{4-}$ octahedra, and β is the X-Pb-X bond angle between neighboring $[PbX_6]^{4-}$ octahedra in the equatorial plane (see **Figure S4a**). The meaning and derivation of each term is discussed below.

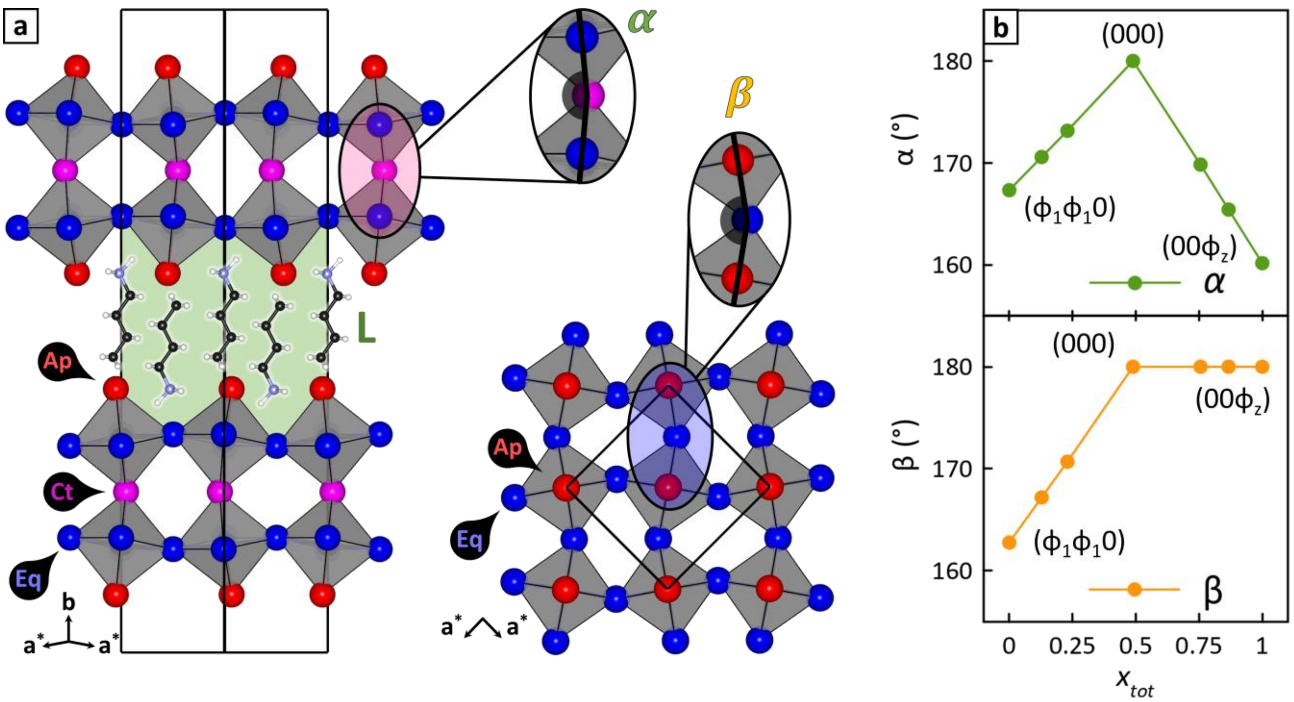

Figure S4. RP structure geometric model. a) Two different views of the RP unit cell, highlighting key features of the model. Apical, Equatorial and Central sites are indicated by black labels (Ap, Eq, and Ct). The interlayer distance, *L*, is shown in green. Insets show the α and β bond angles. b) Modeled dependence of α and β on x_{tot}. Aleksandrov notation shown for the transition structure at x_{tot} = 0.5 and for corresponding pure-halide limiting structures.

1) Fractional composition of halide X sites in the RP unit cell.

Bilayer RP structures feature three different kinds of halide (X) sites: Apical (Ap), Equatorial (Eq) and Central (Ct). The total number of X sites per formula unit is 7, as indicated by the stoichiometry $(BA)_2MAPb_2X_7$. Of these, 2 are Apical, 4 are Equatorial and 1 is Central. **Equation S3** relates the total halide sample composition (x_{tot}), measured by SEM-EDXS, with that of each X site weighted by their multiplicity:

$$x_{\mathrm{tot}} = \frac{2}{7}x_{\mathrm{Ap}} + \frac{4}{7}x_{\mathrm{Eq}} + \frac{1}{7}x_{\mathrm{Ct}}. \quad \textbf{(S3)}$$

Central sites closely resemble those found in 3D-$APbX_3$ perovskites, and hence are most likely to be occupied by I^- and Br^- with little or no preference. It is therefore assumed that $x_{\mathrm{Ct}} \approx x_{\mathrm{tot}}$.

2) [Pb-X] bond lengths as functions of X site composition.

[Pb-X] bond lengths are linear combinations of [Pb-Br] and [Pb-I] bond lengths, weighted by the composition of respective halide sites:

$$[\mathrm{Pb-X}]_{\mathrm{Eq}} = x_{\mathrm{Eq}}[\mathrm{Pb-Br}]_{\mathrm{Eq}} + (1 - x_{\mathrm{Eq}})[\mathrm{Pb-I}]_{\mathrm{Eq}} \quad \textbf{(S4)}$$

$$[\mathrm{Pb-X}]_{\mathrm{Ap}} = x_{\mathrm{Ap}}[\mathrm{Pb-Br}]_{\mathrm{Ap}} + (1 - x_{\mathrm{Eq}})[\mathrm{Pb-I}]_{\mathrm{Ap}} \quad \textbf{(S5)}$$

$$[\mathrm{Pb-X}]_{\mathrm{Ct}} = x_{\mathrm{Ct}}[\mathrm{Pb-Br}]_{\mathrm{Ct}} + (1 - x_{\mathrm{Ct}})[\mathrm{Pb-I}]_{\mathrm{Ct}} \quad \textbf{(S6)}$$

Table S2 provides SCXRD-measured values for [Pb-Br] and [Pb-I] bond lengths in pure-halide $(BA)_2MAPb_2Br_7$ and $(BA)_2MAPb_2I_7$.

Table S2. SCXRD [Pb-Br] and [Pb-I] bond lengths.

**SCXRD Data**	**[Pb-Br]**	**[Pb-I]**
Ap	2.914 Å*	3.083 Å
Eq**	2.992 Å	3.173 Å
Ct	3.064 Å	3.267 Å

** [Pb-Br]$_{Ap}$ is calculated between Pb and the average position of two disordered Br sites, see (BA)2MAPb2I7.cif*

*** [Pb-X]$_{Eq}$ values are the average of the four equatorial bond lengths*

3) Bond angles α and β

The bond angles α and β depend on the octahedra tilting mode adopted by the RP structure. The two pure-halide compounds, $(BA)_2MAPb_2Br_7$ and $(BA)_2MAPb_2Br_7$, exhibit different tilting modes [$(00\Phi_z)$ and $(\Phi_1\Phi_1 0)$, respectively]. It is assumed that x_{tot} = 0.5 represents a sharp transition between these tilting modes, with x_{tot} = 0.5 adopting a no-tilting (000) structure.

Angles α and β are therefore assumed to vary linearly within the intervals $0 \leq x_{tot} < 0.5$ and $0.5 \leq x_{tot} \leq 1$, and can be expressed as the following weighted sums (**Equations S7-S8**). **Figure S4b** shows resulting α and β values as x_{tot} increases from 0 to 1.

$$\alpha = f\alpha_{\mathrm{I}} + (1-f)\alpha_{x=0.5} \quad (x<0.5) \quad \textbf{(S7a)}$$

$$= (1-f)\alpha_{x=0.5} + f\alpha_{\mathrm{Br}} \quad (x\geq 0.5) \quad \textbf{(S7b)}$$

$$\beta = f\beta_{\mathrm{I}} + (1-f)\beta_{x=0.5} \quad (x<0.5) \quad \textbf{(S8a)}$$

$$= (1-f)\beta_{x=0.5} + f\beta_{\mathrm{Br}} \quad (x\geq 0.5) \quad \textbf{(S8b)}$$

$$f = \frac{0.5 - x_{\mathrm{tot}}}{0.5} \quad \textbf{(S9)}$$

Equation S9 describes f as the Br fraction in the sample, compared to the tilting mode transition value of x_{tot} = 0.5. Limiting α and β values for $(BA)_2MAPb_2Br_7$ and $(BA)_2MAPb_2I_7$ have been measured by SCXRD and are reported in **Table S3**.

Table S3. α and β values for $(BA)_2MAPb_2Br_7$ and $(BA)_2MAPb_2I_7$

**Angle**	**$(BA)_2MAPb_2Br_7$**	**$(BA)_2MAPb_2I_7$**
α	160.2°	167.3°
β	180.0°	162.8°

4) Estimation of L as a function of a^* and x_{Ap}

The distance L between two neighboring $[PbX_6]^{4-}$ bilayers, measured at the Pb^{2+} ion plane level, is established as follows. The space between bilayers is considered occupied by neutral L-X ligands, whose volume $V_{L\text{-}X} = V_L + V_X$ is the sum of the constant ammonium chain volume V_L and the variable halide volume V_X. Overall, the $V_{L\text{-}X}$ volume is assumed to be a weighted sum of limiting BA-I and BA-Br volumes ($V_{L\text{-}Br}$ and $V_{L\text{-}I}$ respectively):

$$V_{\mathrm{L}} = x_{\mathrm{Ap}}V_{\mathrm{L-Br}} + (1 - x_{\mathrm{Ap}})V_{\mathrm{L-I}}. \quad \textbf{(S10)}$$

Since the volume occupied by L-X ligands is confined vertically by octahedra layers and horizontally by unit cell faces, it can be calculated as:

$$V_{\mathrm{L-X}} = \frac{(a^*)^2 L}{4}. \quad \textbf{(S11)}$$

Where the ¼ factor is due to the presence of four L-X units per $(a^*)^2$ unit area. Terminal $V_{\mathrm{L\text{-}Br}}$ and $V_{\mathrm{L\text{-}I}}$ values are calculated based on SCXRD-determined a^* parameters and L lengths, and are reported in **Table S4**.

Table S4. SCXRD V_L values for $(BA)_2MAPb_2Br_7$ and $(BA)_2MAPb_2I_7$

$V_{\mathrm{L\text{-}Br}}$	$V_{\mathrm{L\text{-}I}}$
234.3 Å^3	261.4 Å^3

Finally, **Equation S11** can be inverted to yield L as a function of a^* (**Equation S12**). This relation can be used to calculate L for mixed-halide samples. Note that L is a function of x_{Ap} through the term $V_{\mathrm{L\text{-}X}}$ as well as a function of x_{Eq} through the term a^*.

$$L = 4 \cdot \frac{V_{\mathrm{L-X}}}{(a^*)^2} \quad \textbf{(S12)}$$

To validate the assumption that $V_L \approx$ constant and $V_{\mathrm{L\text{-}X}}$ only depends on the composition of the Apical site, we compared the volume variation induced by the Br→I replacement in one unit of BA-X (our samples, **Table S4**) and in one unit of NH_4X (published NH_4X structures, NH_4Br = 66.9 Å^3 [ICSD-24916]; NH_4I = 95.7 Å^3 [ICSD-22150]). Replacing Br→I in NH_4X results in ΔV = 28.8 Å^3, that is very close to $\Delta V = V_{\mathrm{L\text{-}I}} - V_{\mathrm{L\text{-}Br}}$ = 27.1 Å^3 measured for our samples. This demonstrates that the volume variation can be entirely attributed to the halide replacement and, therefore, that the volume occupied by ammonium cations remains unchanged.

We also compared the $V_{\mathrm{L\text{-}X}}$ values in n = 2 $(BA)_2MAPb_2X_7$ structures (our samples, **Table S4**) and in n = 1 $(BA)_2PbX_4$ structures (published: COD-1545801; COD-2102938). These are remarkably close for both bromine-based ($V_{\mathrm{L\text{-}Br}}$ = 236.6 Å^3 [n=1] vs 234.4 Å^3 [n=2]) and iodine-based compounds ($V_{\mathrm{L\text{-}I}}$ = 266.2 Å^3 [n=1] vs 261.4 Å^3 [n=2]), even if the compared structures feature drastically different stoichiometries and adopt different octahedra tilting modes. Moreover, for n = 1 structures the Br→I replacement results in ΔV = 29.7 Å^3, again fully compatible with the examples discussed above. This further demonstrates that considering constant the volume V_L occupied by ammonium cations is a robust and reliable approximation.

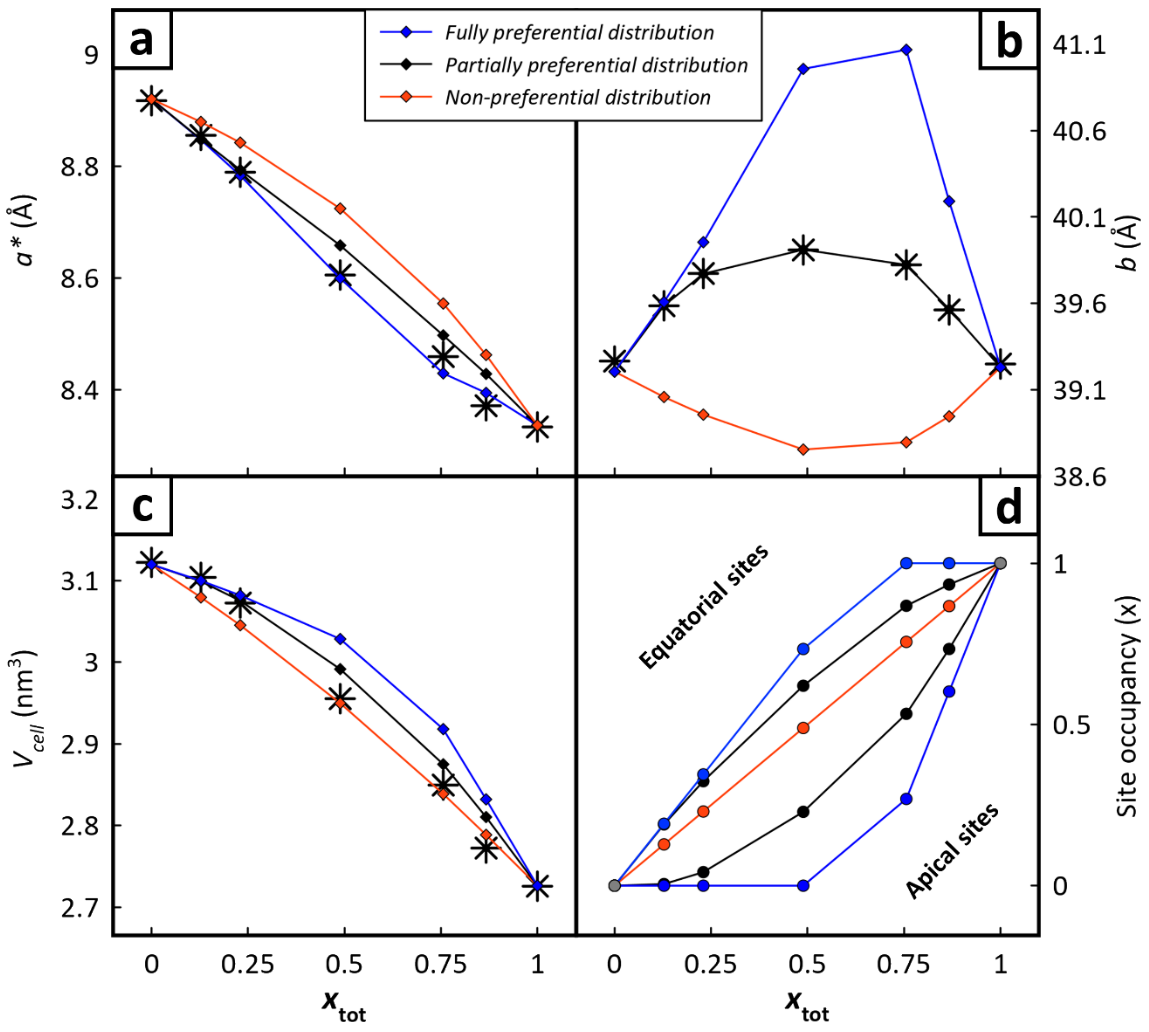

Figure S5. Fully preferential and non-preferential halide distribution limit cases. Simulated lattice parameters *a** (panel a), *b* (panel b), unit cell volume *V* (panel c) and fractional halide composition of Apical and Equatorial halide sites (panel *d*) for two limit cases: fully preferential distribution of halides (blue lines) and non-preferential distribution of halides (red lines). Black lines reproduce the case of a partially preferential distribution of halides, as shown in **Figure 2** of the Main Text. Black asterisks represent parameters measured experimentally.

Single-crystal X-ray diffraction results

The structure models obtained from SCXRD data are provided as .cif files: (BA)2MAPb2I7.cif, (BA)2MAPb2I7.cif, and (BA)2MAPb2(Br0.5I0.5)7.cif for pure bromine, pure iodine, and x = 0.5 mixed bromine/iodine RP perovskites, respectively. Data have been recorded at room temperature to allow direct comparisons with room temperature PXRD data. We observe that cooling RP specimens to cryogenic temperatures results in lattice deformations, occasionally accompanied by fracturing.

In general, structures were solved by dual-space methods, and expanded and refined routinely from difference Fourier maps. Due to disorder of alkyl amines, in all three structures the organic cations have been modeled using isotropic displacement parameters. In contrast, Pb, I, and Br atoms have all been modeled using anisotropic displacement parameters. Hydrogen atoms could not be located or geometrically placed on

disordered MA cations. They have, however, been included in the chemical formula for completeness. Hydrogen atoms bonded to BA cations have been modeled in an idealized geometry. In all studied cases, the BA cations are found to be disordered in the interlayer space. The proposed models best account for the electron density therein.

The structure of $(BA)_2MAPb_2Br_7$ has been refined with a racemic twin component, yielding a 0.548:0.452 domain ratio. $(BA)_2MAPb_2I_7$ has likewise been modeled with a racemic twin component, yielding a 0.51:0.49 twin ratio. In both cases, the presence of an inversion center was contraindicated by attempts to model the structures in centrosymmetric space groups and by the lack of missed inversion symmetry in the asymmetric unit. Mild restraints were applied to the bond distances, angles, and displacement parameters of BA and MA cations in all structures. For the mixed-halide $(BA)_2MAPb_2(Br_xI_{1-x})_7$, halogen positions have been refined with Br^- and I^- occupying the same site. Standard equivalencies to constrain atom positions and displacement parameters were employed.

Site occupancies were refined to account for measured electron densities with Br and I fractions adding up to unity. Site compositions were assumed independent of each other. This yielded an overall halide ratio of x_{tot} = 0.44, with anions distributed as follows:

Table S5. Halide composition of Ap, Eq, and Ct sites as determined by electron density.

**Site**	**Br %**	**I %**
Ap	7	93
Eq	64	36
Ct	37	63
Total	44	56

For a better comparison with the developed geometric model, site occupancies have also been estimated based on bond lengths by applying **Equations S4-6**. This yields an overall halide ratio of x_{tot} = 0.56, with anions distributed as follows:

Table S6. Halide composition of Ap, Eq, and Ct sites as determined by bond lengths.

**Site**	**Br %**	**I %**
Ap	29	71
Eq	74	26
Ct	42	58
Total	56	44

Spin coated thin films

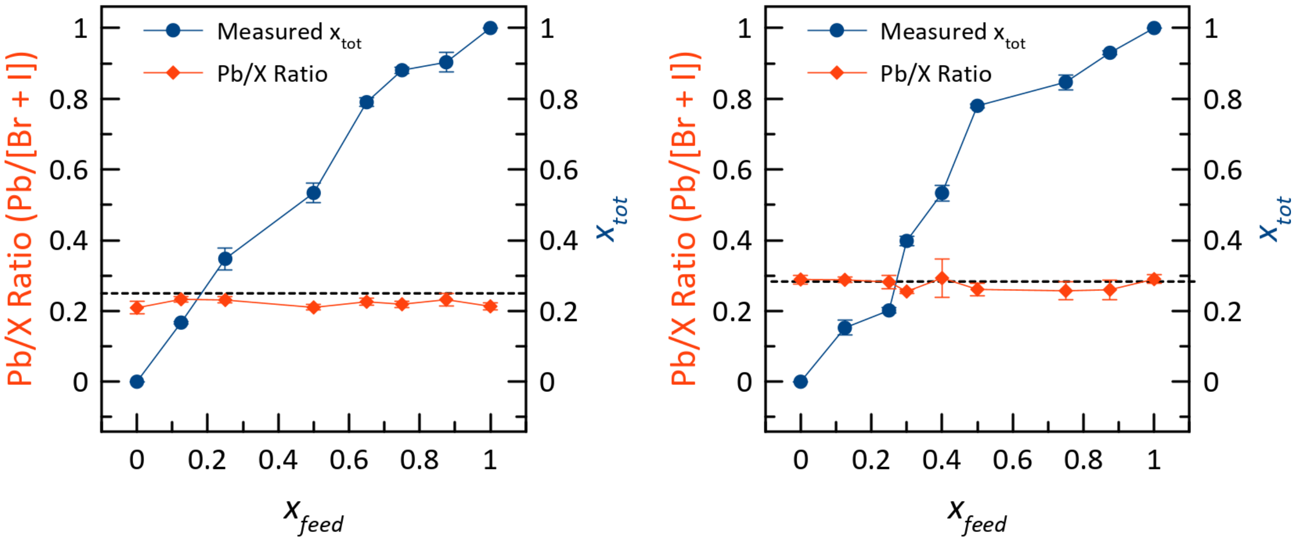

Figure S6. Composition calibration curves for spin coated thin films. a) Calibration curve for n = 1 **(**$(BA)_2Pb(Br_xI_{1-x})_4$ films. b) Calibration curve for n = 2 $(BA)_2MAPb_2(Br_xI_{1-x})_7$ films. Blue traces: experimental halide composition plotted versus halide precursor feed ratio. Red traces: experimental Pb/X ratio as measured by SEM-EDXS. In both scales, x = Br/[Br+I] where Br and I are the atomic fractions of bromine and iodine introduced in precursor solutions (feed ratio) and measured experimentally by SEM-EDXS (measured). Solid blue and red lines are guides to the eye. Horizontal, dashed black lines represent the ideal Pb/X values of 1/4 = 0.25 for n = 1 and 2/7 = 0.286 for n = 2, established from the stoichiometry of $(BA)_2MAPb_2X_7$ and $(BA)_2MAPb_2(Br_xI_{1-x})_7$ respectively. These lines serve as controls to assess the reliability of SEM-EDXS analyses.

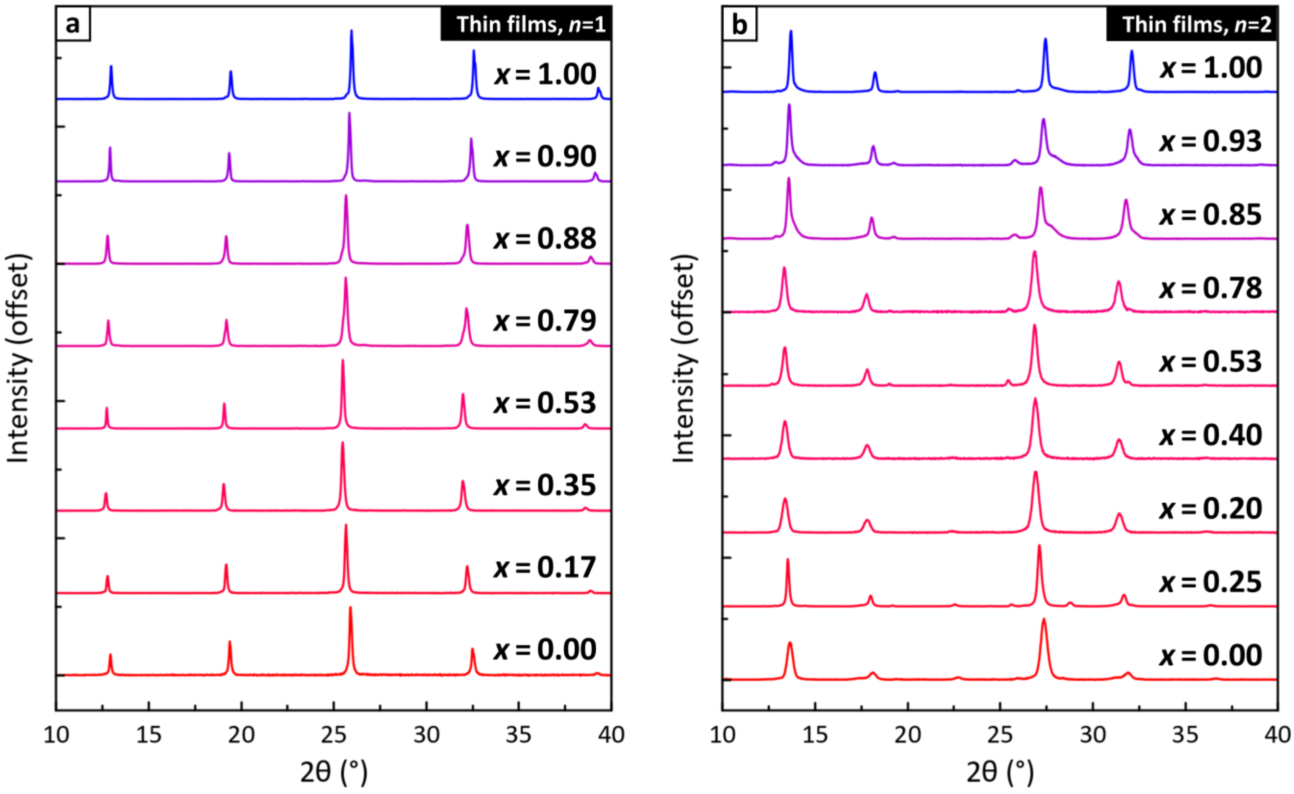

Figure S7. XRD patterns of spin coated thin films. a) n = 1 $(BA)_2Pb(Br_xI_{1-x})_4$ and b) n = 2 $(BA)_2MAPb_2(Br_xI_{1-x})_7$ films.
